# N-Terminal Regions of Prion Protein: Functions and Roles in Prion Diseases

**DOI:** 10.3390/ijms21176233

**Published:** 2020-08-28

**Authors:** Hideyuki Hara, Suehiro Sakaguchi

**Affiliations:** Division of Molecular Neurobiology, The Institute for Enzyme Research (KOSOKEN), Tokushima University, 3-18-15 Kuramoto, Tokushima 770-8503, Japan; hara@tokushima-u.ac.jp

**Keywords:** prion protein, prion, prion disease, neurodegeneration, protein conformation

## Abstract

The normal cellular isoform of prion protein, designated PrP^C^, is constitutively converted to the abnormally folded, amyloidogenic isoform, PrP^Sc^, in prion diseases, which include Creutzfeldt-Jakob disease in humans and scrapie and bovine spongiform encephalopathy in animals. PrP^C^ is a membrane glycoprotein consisting of the non-structural *N*-terminal domain and the globular C-terminal domain. During conversion of PrP^C^ to PrP^Sc^, its 2/3 C-terminal region undergoes marked structural changes, forming a protease-resistant structure. In contrast, the N-terminal region remains protease-sensitive in PrP^Sc^. Reverse genetic studies using reconstituted PrP^C^-knockout mice with various mutant PrP molecules have revealed that the N-terminal domain has an important role in the normal function of PrP^C^ and the conversion of PrP^C^ to PrP^Sc^. The N-terminal domain includes various characteristic regions, such as the positively charged residue-rich polybasic region, the octapeptide repeat (OR) region consisting of five repeats of an octapeptide sequence, and the post-OR region with another positively charged residue-rich polybasic region followed by a stretch of hydrophobic residues. We discuss the normal functions of PrP^C^, the conversion of PrP^C^ to PrP^Sc^, and the neurotoxicity of PrP^Sc^ by focusing on the roles of the N-terminal regions in these topics.

## 1. Introduction

Conformational conversion of the normal cellular isoform of prion protein, designated PrP^C^, to the abnormally folded, amyloidogenic isoform, PrP^Sc^, is a key pathogenic event in prion diseases, a group of fatal neurodegenerative disorders that include Creutzfeldt–Jakob disease (CJD) in humans, scrapie in sheep, bovine spongiform encephalopathy (BSE) in cattle, and chronic wasting disease in deer [1,2,3,4]. These diseases are pathologically characterized by neuronal cell loss, spongiform degeneration, gliosis, and PrP^Sc^ accumulation in the brain [5]. Prions, or proteinaceous infectious particles, are the causative agents of these diseases [6,7]. It is believed that prions consist of, if not entirely, PrP^Sc^ molecules, and catalyze conformational conversion of PrP^C^ to PrP^Sc^ through a seeded protein polymerization mechanism, eventually propagating PrP^Sc^ or prions themselves [6,7]. Indeed, it has been shown that mice devoid of PrP^C^ (*Prnp^0/0^*) are resistant to prion infection, neither propagating prions nor PrP^Sc^ in their brains nor developing disease even after intracerebral inoculation with prions [8,9,10,11]. 

**Figure 1:**
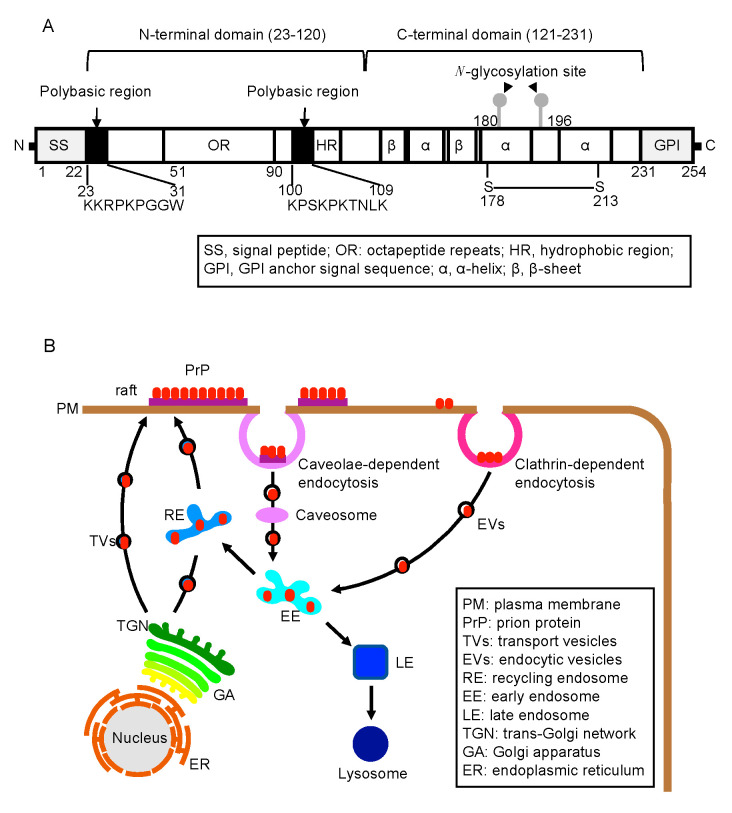
Structure and biosynthesis of PrP^C^. (**A**) Structural configuration of PrP^C^. Arabic numbers indicate positions of amino acids. (**B**) Biosynthetic pathways of PrP^C^, including the vesicle transport pathway from the ER to the plasma membrane, particularly raft domains, and the clathrin- or caveolae-dependent endocytic pathway, which connects to recycling pathway or degradation pathway to lysosomes.

PrP^C^ is a highly conserved, glycosylphosphatidylinositol (GPI)-anchored membrane glycoprotein among mammalian species [12]. It is expressed most abundantly in the central nervous system, particularly by neurons, and to a lesser extent in other non-neuronal tissues, such as the lymphoreticular system, lung, and kidney [13]. PrP^C^ consists of two domains; the highly flexible, nonstructural N-terminal (residues 23–120) and the globular C-terminal (residues 121–231) [14,15,16] (Figure 1A). The globular C-terminal domain is composed of three α-helices and two short anti-parallel ß-sheets. Upon conversion to PrP^Sc^, PrP^C^ undergoes marked structural changes in its 2/3 C-terminal region to form a proteinase K (PK)-resistant structure, while most regions of the N-terminal domain remain PK-sensitive [13]. Reverse genetic studies using reconstituted *Prnp^0/0^* mice and various mutant PrP molecules have revealed that the N-terminal domain has an important role not only in the normal function of PrP^C^ but also in the conversion of PrP^C^ to PrP^Sc^. The N-terminal domain includes several characteristic regions, such as the so-called polybasic region (residues 23–31), which is rich in positively charged residues, the octapeptide repeat (OR) region (residues 51–90) consisting of five repeats of an octapeptide sequence, and the post-OR region (residues 91–120) including the second polybasic region followed by a stretch of hydrophobic amino acid residues [1,2,3,4] (Figure 1A). Here we discuss the role of each N-terminal region in the normal function of PrP^C^, the conversion of PrP^C^ to PrP^Sc^, and the neurotoxicity of PrP^Sc^.

## 2. The N-Terminal Domain in the Function of PrP^C^

### 2.1. Biosynthesis of PrP^C^

The gene for PrP^C^, termed *Prnp*, in human and mouse consists of 2 and 3 exons and resides on chromosome 20 and 2, respectively. The protein coding sequence lies within the last single exon [17,18]. PrP^C^ is synthesized as a precursor protein in the endoplasmic reticulum (ER). The N-terminal and C-terminal sequences, which are rich in hydrophobic residues, are removed as a signal peptide sequence and a GPI-anchor signal sequence, respectively, in the ER (Figure 1A) [17,18]. PrP^C^ also undergoes several post-translational modifications en route to the cell surface, including a GPI anchor attachment at the C-terminus, *N*-glycosylation at two sites, and formation of a disulfide bond in the C-terminal domain (Figure 1A) [19,20,21,22,23,24]. On the cell surface, PrP^C^ is predominantly localized at the so-called “raft” domains and constitutively internalized via clathrin- and caveolae-dependent endocytosis (Figure 1B) [25,26,27]. Some of the internalized PrP^C^ molecules are recycled to the cell surface and others are trafficked to lysosomes for degradation (Figure 1B) [28,29].

Copper is known to bind to the OR region and induce the clathrin-dependent internalization of PrP^C^ [30]. It has been suggested that copper binding could cause conformational changes in the OR region and thereby dissociate PrP^C^ from conjectural molecules located at raft domains, and that dissociated PrP^C^ then moves to non-raft domains, where it interacts with other conjectural non-raft molecules through the N-terminal polybasic region to be endocytosed via clathrin-coated vesicles [30]. We have shown that sortilin, a type 1 glycoprotein in the vacuolar protein sorting 10 protein family, interacts with the N-terminal domain of PrP^C^ and functions as a sorting receptor for lysosomal degradation of PrP^C^ [31]. Sortilin also interacts with PrP^Sc^ and facilitates its lysosomal degradation [31]. We also have shown that sortilin-knockout mice develop prion disease with shorter incubation times and rapid brain accumulation of PrP^Sc^ after inoculation with prions, compared to control wild-type (WT) mice [31], suggesting that the sortilin-mediated trafficking of PrP^C^ and PrP^Sc^ to lysosomes could be a host defense mechanism in prion diseases. Low-density lipoprotein receptor-related protein 1 has also been reported as a cargo receptor for PrP^C^ for transport from the Golgi apparatus to the cell surface and from the cell surface to endosomes [32].

### 2.2. Various Abnormal Phenotypes Are Spontaneously Observed in Prnp^0/0^ Mice

*Prnp^0/0^* mice are born with no obvious defects, indicating that PrP^C^ could be dispensable for embryonic development [11,33,34]. However, various neurophysiological and neuropathological abnormalities have been reported in *Prnp^0/0^* mice, including poor performance in certain behavioral tests [35], impaired long-term potentiation (LTP) in the hippocampal CA1 neurons [36], altered sleep and circadian rhythms [37], demyelination in spinal cords and peripheral nerves [38], and abnormal olfactory function [39,40]. These results suggest that PrP^C^ is involved in various neuronal functions. However, normal LTP in *Prnp^0/0^* mice has been reported by other investigators [41].

### 2.3. The OR Region in the Cell-Protective Role of PrP^C^

We and others have shown that *Prnp^0/0^* mice are vulnerable to ischemic brain, heart, or kidney damage, displaying higher apoptotic cell death and higher oxidative stress in the damaged tissues [42,43,44,45,46]. We also recently reported that *Prnp^0/0^* mice are highly sensitive to infection with influenza A viruses (IAVs), showing higher morbidity and mortality with higher inflammation, higher apoptotic cell death, and higher oxidative stress in their lungs [47]. Treatment with a scavenger for reactive oxygen species (ROS) or an inhibitor for ROS-generating xanthine oxidase rescued *Prnp^0/0^* mice from lethal IAV infection [47]. In contrast, PrP molecules lacking the OR region failed to protect *Prnp^0/0^* mice from lethal IAV infection and ischemic brain damage [47,48]. These results suggest that PrP^C^ could play a cell-protective role against oxidative stress through the OR region. The OR region is known to bind copper [49]. Indeed, the copper content and enzymatic activity of copper/zinc-dependent superoxide dismutase (SOD) were lower in *Prnp^0/0^* lungs and brains than in control WT tissues [47,49]. It is thus possible that PrP^C^ could function as a transporter of the OR region-bound copper to copper/zinc-SOD, thereby regulating enzyme activity and eventually protecting from oxidative stress. It was reported that PrP^C^ itself might have SOD-like activity [50]. However, other investigators have failed to detect SOD activity in PrP^C^ in vitro and in vivo [51,52].

The OR region is also suggested to be involved in other cell-protective mechanisms of PrP^C^. Overexpression of PrP^C^, but not an OR-lacking PrP molecule, was shown to protect against Bax-mediated apoptosis in human primary neurons [53], suggesting that PrP^C^ could function as an anti-apoptotic molecule through the OR region. Oh et al. also reported that autophagy was activated in *Prnp^0/0^* hippocampal neuronal cultured cells under serum deprivation, and that expression of PrP^C^ prevented the activation of autophagy in the cells, but an OR-deleted PrP mutant did not [54], suggesting that PrP^C^ could regulate autophagy activity in neuronal cells through the OR region. It remains to be determined if these functions of PrP^C^ are attributable to the activation of copper/zinc-SOD.

### 2.4. The Polybasic Region in the Function of PrP^C^

The polybasic region is also suggested to be involved in the anti-oxidative activity of PrP^C^. Oxidative stress was shown to enhance cleavage of PrP^C^, releasing the N-terminal fragment, termed N2, which encompasses residues 23–89 including the polybasic region [55], and the N2 fragment protected neuronal cells against oxidative stress through stimulation of MEK1 signaling [56]. Two proline residues in the polybasic region were shown to be important for the N2-mediated anti-oxidative activity [55]. Other roles have also been reported for the polybasic region including that it is involved in mediating the interaction of PrP^C^ with tubulin or glycosaminoglycan [57,58,59,60], the ß-secretase-mediated cleavage of the Alzheimer’s amyloid precursor protein [61], and DNA repair [62].

## 3. The N-Terminal Domain of PrP^C^ in Prion Disease

### 3.1. The Polybasic Region in Prion Disease

Reconstituted *Prnp^0/0^* mice by transgenic introduction of a mutant PrP with a deletion of the polybasic region residues 23–31, designated Tg(PrP∆23–31)/*Prnp^0/0^* mice, were shown to develop prion disease with markedly elongated incubation times and delayed accumulation of PrP^Sc^∆23–31 in their brains after inoculation with RML scrapie prions (Table 1) [63]. PrP^Sc^∆23–31 accumulated in the brains of Tg(PrP∆23–31)/*Prnp^0/0^* mice showed similar resistance to PK to WT PrP^Sc^ [63], suggesting that the polybasic region does not affect the PK-resistance of PrP^Sc^. These results suggest that the polybasic region could play a crucial role in the pathogenesis of prion diseases. We have shown that Tg(PrP∆25–50)/*Prnp^0/0^* mice developed disease without elongated incubation times after infection with RML and 22L prions (Table 1) [64], suggesting that the remaining residues 23 and 24 in PrP∆25–50 could be enough for the polybasic region to support prion pathogenesis. However, it was reported that incubation times were only slightly longer or not elongated at all in Tg(PrP∆23–26)/*Prnp^0/0^* mice after infection with 127S and LA19K scrapie prions and BSE prions (Table 1) [65]. PrP∆23–26 includes intact residues 27–31, but lacks residues 23 and 24 in the polybasic region. It is thus possible that the polybasic region might require that both residues 23–24 and 27–31 are intact to fully support prion pathogenesis [64]. Consistent with this idea, mutations of lysine residues at positions 24 and 27 together with a mutation of an arginine residue at position 25 rendered ovine PrP highly resistant to 127S and LA19K scrapie prions and BSE prions (Table 1) [65]. We also showed that *Prnp^0/0^* mice transgenic for mouse PrP with substitutions of lysine residues at positions 23, 24, and 27 to alanine residues, or PrP3K3A, markedly reduced their susceptibility to RML and 22L scrapie prions (Table 1) [66], suggesting that positively charged residues in residues 23–24 and 27–31 could be important for the polybasic region to support prion pathogenesis. No PK-resistant PrP3K3A was spontaneously produced in the brain of uninfected Tg(PrP3K3A)/*Prnp^0/0^* mice [66], suggesting that mutations in the polybasic region might not cause structural changes in mutant PrPs.

### 3.2. The OR Region in Prion Disease

Insertion of various numbers of an OR sequence, ranging from one to nine, and deletion of one OR sequence in the OR region have been identified in patients with hereditary CJD [67]. Brain homogenates from patients with five, seven, or eight extra OR sequences in PrP can transmit the disease to animals after intracerebral inoculation [68]. This suggests that disruption of the integrity of the OR region by the insertion or deletion of the OR sequence could cause structural instability of mutated PrPs, ultimately leading to their spontaneous conversion to pathogenic, infectious PrPs. We failed to detect PK-resistant PrP in the brains of Tg(PrPΔOR)/*Prnp^0/0^* mice, which express PrP with a deletion of the OR region alone (Table 2) [69,70], suggesting that spontaneous conversion of mutated PrPs with extra OR sequences to PK-resistant PrPs could be due to gain-of-function, but not due to loss-of-function, of the mutated OR region. Consistent with this, Tg(PG14)/*Prnp^0/0^* mice, which express a PrP mutant with nine extra OR sequences in the OR region, developed spontaneous cerebellar neurodegeneration including granule cell death, with very slight but substantial accumulation of PK-resistant PrP^Sc^PG14 in their brains (Table 2) [71,72]. However, PrP^Sc^PG14 had no prion infectivity in animal bioassays (Table 2) [73]. Also, transgenic expression of bovine PrP with four extra OR sequences, or bo10OR-PrP, caused a slowly progressive neurological disorder with ataxia, vacuolization, gliosis, and cerebellar granule cell loss in *Prnp^0/0^* mice (Table 2) [74]. Insoluble and slightly PK-resistant 10OR-PrP^Sc^ molecules accumulated in their brains, but no prion infectivity was found associated with the insoluble 10OR-PrP^Sc^ (Table 2) [74]. These results indicate that PrPPG14 and bo10OR-PrP spontaneously convert to PrP^Sc^PG14 and 10OR-PrP^Sc^, respectively, with structural features shared with PrP^Sc^ that are responsible for the neurotoxicity but not prion infectivity. These results also suggest that the structural features of PrP^Sc^ that contribute to its neurotoxicity and prion infectivity are not identical.

The OR region is also involved in prion infection. We have shown that Tg(PrPΔOR)/*Prnp^0/0^* mice are highly resistant to BSE prions (Table 2) [70]. They developed the disease with markedly elongated incubation times with delayed accumulation of PrP^Sc^ΔOR in their brains after inoculation with BSE prions (Table 2) [70]. Consistent with our results, an increasing number of OR insertions contrarily enhances BSE pathogenesis in mice. *Prnp^0/0^* mice expressing bovine PrP with one extra OR sequence had shortened incubation times when compared with *Prnp^0/0^* mice expressing WT bovine PrP, or bo6OR-PrP, after infection with BSE prions (Table 2) [75]. BSE-inoculated Tg(bo10OR-PrP)/*Prnp^0/0^* mice were also shown to have further shortened incubation times when compared to BSE-inoculated Tg(bo6OR-PrP)/*Prnp^0/0^* mice (Table 2) [74]. These results suggest that the OR region could play a crucial role in BSE prions during the conversion of PrP^C^ to PrP^Sc^. In contrast, Tg(PrPΔOR)/*Prnp^0/0^* mice remained susceptible to RML and 22L scrapie prions, developing the disease without elongated incubation times with slightly less PrP^Sc^ΔOR in their brains after infection with RML and 22L prions (Table 2) [70], suggesting that the OR region might be involved in prion pathogenesis in a strain-dependent manner. However, *Prnp^0/0^* mice expressing PrP with histidine residues in the OR region replaced by glycine residues, termed PrP(TetraH>G), showed significantly prolonged incubation times after infection with RML prions (Table 2) [76]. Further studies are needed to clarify whether or not the OR region might mediate strain-dependent prion pathogenesis.

### 3.3. The Post-OR Region in Prion Diseases

Three mutations in the post-OR region, including P102L (substitution of a proline residue to a leucine residue at position 102), P105L (substitution of a proline residue to a leucine residue at position 105), and A117V (substitution of an alanine residue to a valine residue at position 117), are associated with inherited human prion diseases [67], suggesting that the post-OR region also plays a role in prion diseases. Tg(PrP-P101L) mice, which express high levels of mouse PrP-P101L, the analogous mutation to human PrP-P102L, have been shown to spontaneously develop prion disease-like diseases, with amyloid plaques, spongiform degeneration, and gliosis in their brains (Table 3) [77]. Brain homogenates from ill Tg(PrP-P101L) mice transmitted the disease to 40% of Tg(PrP-P101L) mice, which never spontaneously developed disease due to lower expression of the mutant protein, and 10% of hamsters, but not to WT CD-1 mice, after intracerebral inoculation (Table 3) [78], indicating that PrP^Sc^-P101L could be infectious. Tg mice expressing mouse PrP-A116V (the human homologue of PrP-A117V) at six times the endogenous levels of PrP^C^ also spontaneously developed progressive ataxia with vacuolation and PrP amyloid plaques in their brains (Table 3) [79]. The PrP molecules from Tg(PrP-A116V) brains were partly insoluble and weakly protease-resistant (Table 3) [79]. No data are available regarding whether PK-resistant PrP-A116V is infectious.

The post-OR region could be also involved in prion infection. Tg(PrP∆32–80)/*Prnp^0/0^* mice developed disease without elongation in incubation times and accumulated PrP^Sc^∆32–80 in their brains after infection with RML prions (Table 3) [80], suggesting that residues 32–80 are dispensable for PrP^C^ to convert to PrP^Sc^ after prion infection. However, Tg(PrP∆32–93)/*Prnp^0/0^* mice, which express PrP with a deletion extending to the post-OR region at position 93 from the OR region at position 88, developed disease with longer incubation times and with lower levels of infectivity and PrP^Sc^∆32–93 in their brains after infection with RML prions (Table 3) [81]. Moreover, PrP with a deletion further extending to the post-OR region at position 106, or PrP∆32–106, neither converted to PrP^Sc^ nor supported prion pathogenesis in *Prnp^0/0^* mice after intracerebral inoculation with RML prions (Table 3) [82]. These results suggest that the post-OR residues 91–106, which are completely deleted in PrP∆32–106 and partially in PrP∆32–93, but intact in PrP∆32–80, could have a crucial role in prion infection. However, it remains to be determined if the resistance of Tg(PrP∆32–106)/*Prnp^0/0^* mice to RML prions could be due to deletion of the post-OR residues 91–96 alone or together with deletion of other residues.

## 4. The N-Terminal Domain in Conversion of PrP^C^ to PrP^Sc^

**Figure 2:**
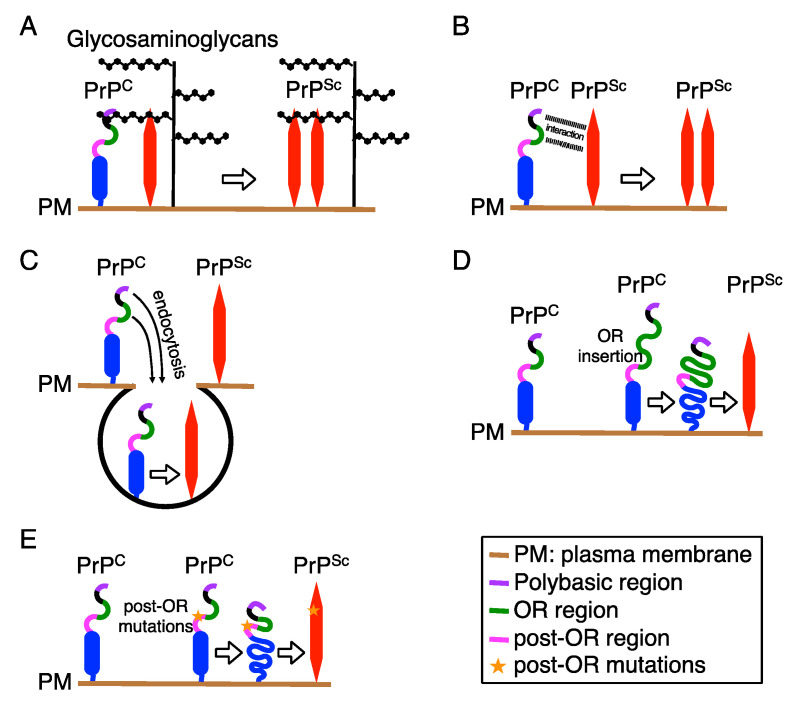
Possible roles of the N-terminal regions in the conversion of PrP^C^ into PrP^Sc^. Upon conversion of PrP^C^ into PrP^Sc^, PrP^C^ might interact with PrP^Sc^ through glycosaminoglycans (**A**) or through the polybasic and OR regions (**B**). (**C**) The polybasic and OR regions are also involved in endocytosis of PrP^C^ to endosomal compartments, where PrP^C^ is considered to convert into PrP^Sc^. Extra OR sequences in the OR region (**D**) and point mutations in the post-OR region (**E**) might render mutated PrPs structurally unstable, ultimately leading to their spontaneous conversion to pathogenic PrPs.

The first step for conversion of PrP^C^ to PrP^Sc^ is an intermolecular interaction between both molecules. The polybasic region has been suggested to be involved in the binding of PrP^C^ and/or PrP^Sc^ to the extracellular matrix proteins glycosaminoglycans through the positively charged residues [58,59,60]. It is thus possible that the polybasic region might promote interaction between PrP^C^ and PrP^Sc^ by recruiting both molecules to glycosaminoglycans, thereby supporting conversion of PrP^C^ to PrP^Sc^ (Figure 2A). The polybasic region has also been suggested to mediate a direct interaction between PrP^C^ and PrP^Sc^, thereby promoting the conversion of PrP^C^ to PrP^Sc^ [63] (Figure 2B).

The next step for conversion is a structural unfolding of the interacting PrP^C^. PrP^C^ is rich in α-helix structures and soluble in non-ionic detergents [83]. In contrast, PrP^Sc^ is abundant in ß-sheet structures and insoluble in non-ionic detergents, forming fibrils [83], suggesting that structural transition of α-helices to ß-sheets in PrP^C^ is an underlying mechanism of the conversion to PrP^Sc^. Several structural models have been proposed for PrP^Sc^ fibrils. The 4-rung ß-solenoid model postulates that a PrP^Sc^ fibril consists of two intertwined protofilaments of PrP^Sc^ molecules [84,85]. In this model, single PrP^Sc^ molecules adopt a solenoid structure of four rungs, each rung including three ß-strands, running perpendicular to fibril axis, stacking each other. The upper and lower ß-solenoid rungs of PrP^Sc^ protofibrils could template an incoming unfolded PrP^C^ molecule to create additional ß-solenoid rungs. Once a new ß-solenoid rung has formed, it continues to template until the unfolded PrP^C^ molecule is completely converted to PrP^Sc^ conformer. In the parallel in-register intermolecular ß-sheet model, single PrP^Sc^ molecules comprise the entire cross-section of a fibril, with many hairpins defined by natural and artificial disulfide bonds [86,87]. They are stacked parallel in-register and perpendicular to the fibril axis by forming intermolecular ß-sheet interactions between them. Endocytic/lysosomal compartments are considered to be a site for conversion of PrP^C^ to PrP^Sc^ [88,89], suggesting that acidic conditions in the endosomal/lysosomal compartments might promote the structural unfolding of PrP^C^. The polybasic and OR regions are involved in endocytosis of PrP^C^ [30,90]. It is thus possible that these regions might play a role in conversion of PrP^C^ to PrP^Sc^ by mediating endocytosis of PrP^C^ to acidic endocytic/lysosomal compartments (Figure 2C). Insertion of extra OR sequences in the OR region or mutations in the post OR region are associated with spontaneous conversion of mutated PrPs to pathogenic PrPs, causing hereditary prion diseases in humans [67], suggesting that structural instability of the OR region or in the post-OR region might also be involved in the unfolding of the mutant PrPs (Figure 2D,E). Indeed, recombinant human PrPs with three or five extra OR sequences have been reported to spontaneously form aggregates [91]. Copper binding to recombinant mouse PrP was reported to cause novel intramolecular interactions, including those between the N-terminal residues 90–120 and the C-terminal residues 144–147 and its nearby residues 139–143, and between the N-terminal region comprising the OR region and the C-terminal residues 174–185 [92], suggesting that copper binding might also be involved in the unfolding of PrP^C^. Copper is able to bind to histidine residues located in the OR and post-OR regions [76]. We have shown that, while Tg(PrP∆OR)/*Prnp^0/0^* mice were highly resistant to BSE prions, they still remained susceptible to RML and 22L prions [70], suggesting that copper binding to histidine residues in the OR region might be irrelevant to the unfolding of PrP^C^. Indeed, it has been shown that histidine residues in the post-OR could be important for conversion of PrP^C^ to PrP^Sc^ in acidic conditions [93].

## 5. The N-Terminal Domain and Neurotoxic PrP Molecules

The neurotoxic mechanism of PrP^Sc^ remains largely unknown. However, there have been several reports of neurotoxic PrP molecules causing prion disease-like neurodegeneration, giving rise to an interesting possibility that these neurotoxic PrP molecules might share their neurotoxic mechanism with PrP^Sc^. In addition to a GPI-anchored extracellular form of PrP^C^, another form of PrP, termed ^Ctm^PrP, has been reported [94]. ^Ctm^PrP is a transmembrane form of PrP, with the N-terminus facing the cytoplasm and the C-terminus exposed extracellularly. Increased hydrophobicity in the post-OR region by mutations that cause residues to become hydrophobic, including the mutation in hereditary prion disease (A117V), increase the ratio of ^Ctm^PrP to total forms of PrP molecules in neuronal cells [94]. Interestingly, transgenic mice expressing these mutant PrPs spontaneously develop prion disease-like neurodegeneration with focal vacuolar degeneration in the neuropil and astrocytic gliosis [94]. Moreover, the ratio of ^Ctm^PrP was also reported to increase in the brains of mice infected with prions [95]. These results suggest that ^Ctm^PrP might be responsible for neurodegeneration in prion diseases. However, ^Ctm^PrP from transgenic mice is not infectious [94].

Other neurotoxic PrP molecules have also been reported. It was shown that *Prnp^0/0^* mice transgenic for PrP with a deletion of the N-terminal residues 32–121 or 32–134, which includes the OR region and a section of the post-OR region, spontaneously developed cerebellar neurodegeneration, with marked granule cell death [96]. Other investigators also showed that *Prnp^0/0^* mice expressing a PrP molecule, designated ΔCR, that harbors a deletion of residues 105–125, developed cerebellar neurodegeneration [97], suggesting that deletion of the post-OR residues 105–125 alone could be responsible for the neurodegeneration in *Prnp^0/0^* mice expressing PrP∆21–121 and PrP∆32–134. Interestingly, the neurotoxicity of these mutant PrPs in *Prnp^0/0^* mice is abrogated by co-expression of WT PrP^C^ [96,97], suggesting that, while the toxic PrP molecules generate a neurotoxic signal, WT PrP^C^ transduces a neuroprotective signal to antagonize the neurotoxic signal of the mutant PrPs. It was shown that, in contrast to PrP∆32–134, PrP∆23–134 was not neurotoxic in *Prnp^0/0^* mice, suggesting that the polybasic region residues 23–31, which remain intact in toxic PrP∆32–134 but not in non-toxic PrP∆23–134, are critical for the neurotoxicity of mutant PrPs [98,99]. Patch-clamp electrophysiological experiments revealed that ΔCR induced abnormal spontaneous ionic currents in various cultured cells and neurons through the polybasic region, and that these currents were suppressed by co-expression of WT PrP^C^ [100,101], suggesting that the abnormal ionic currents might be the neurotoxic signal of the mutant PrPs. It would be thus worthy to investigate whether PrP^Sc^ could generate similar abnormal currents in neurons.

## 6. Conclusions

It has been shown that the non-structural, flexible N-terminal domain, which includes various specific regions such as the polybasic region, OR regions, and post-OR region, has a role in not only the normal function of PrP^C^ but also in the pathogenesis of prion diseases through regulation of the conversion of PrP^C^ to PrP^Sc^ and the neurotoxicity of PrP^Sc^. Further elucidation of the exact mechanism of how each of the N-terminal regions could regulate the normal function of PrP^C^ and prion pathogenesis would be of great help for understanding the function of PrP^C^ and prion pathogenesis, and eventually for developing therapeutics for prion diseases.

## Figures and Tables

**Table 1:** Effects of various mutations in the polybasic region of PrP^C^ on acquired prion diseases.

Disease Type	PrPs	Amino Acid Sequence of the Polybasic Region (Residues 23–31) ^1^	Susceptibility to Prions	References
Acquired prion disease	WT PrP	KKRPKPGGW	• Normal.	
	PrP∆23–31	− − − − − − − − −	• Markedly reduced to RML scrapie prions.	[63]
	PrP∆25–50	KK− − − − − − −	• Not reduced to RML and 22L scrapie prions	[64]
	PrP∆23–26	− − − −KPGGW	• Only slightly or not reduced to 127S and LA19K scrapie prions and BSE prions.	[65]
	PrP-M	KQHPHPGGW	• Markedly reduced to 127S and LA19K prions and BSE prions	[65]
	PrP3K3A	AARPAPGGW	• Markedly reduced to RML and 22L scrapie prions.	[66]

^1^ Amino acids are indicated by single letters. Underline letters indicate amino acids mutated.

**Table 2:** Effects of various mutations in the OR region of PrP^C^ on hereditary and acquired prion diseases.

**Disease Type**	**PrPs**	**Number of the OR Sequence**	**Clinicopathological Features**	**References**
Hereditary prion disease	PG14	14 ^1^	• Spontaneously develop cerebellar neurodegeneration. • Accumulate very slightly but substantially PK-resistant PrP^Sc^PG14 in the brain. • No prion infectivity associated with PrP^Sc^PG14.	[71,72,73]
	Bo10OR-PrP	10 ^2^	• Spontaneously develop cerebellar neurodegeneration. • Accumulate insoluble and slightly PK-resistant 10OR-PrP^Sc^ in their brains. • No prion infectivity associated with 10OR-PrP^Sc^.	[74]
**Disease Type**	**PrPs**	**Number of the OR Sequence**	**Susceptibility to Prions**	**References**
Acquired prion disease	PrP∆OR	0 ^1^	• Reduced to BSE prions, but not to RML and 22L scrapie prions.	[70]
	Bo7OR-PrP	7 ^2^	• Increased to BSE prions.	[75]
	Bo10OR-PrP	10 ^2^	• Increased to BSE prions.	[74]
	PrP(TetraH>G)	5^1^ (with 4 histidine residues mutated to glycine residues)	• Reduced to RML prions.	[76]

^1^ Normal mouse PrP^C^ contains 5 repeats of the OR sequence. ^2^ Normal bovine PrP^C^ contains 6 repeats of the OR sequence.

**Table 3:** Effects of various mutations in the post-OR region of PrP^C^ on hereditary and acquired prion diseases.

**Disease Type**	**PrPs**	**The Post-OR Sequence**	**Clinicopathological Features**	**References**
Hereditary prion disease	PrP-P101L	Proline residue at position 101 mutated to leucine residue in mouse PrP	• Spontaneously develop prion disease-like diseases. • Accumulate weakly protease-resistant PrP-P101L in the brain. • Accumulate prion infectivity associated with weakly protease-resistant PrP-P101L.	[77,78]
	PrP-A116V	Alanine residue at position 116 mutated to valine residue in mouse PrP	• Spontaneously developed prion disease-like diseases. • Accumulate partly insoluble and weakly protease-resistant PrP-A116V in the brain. • No data available as to infectivity associated with protease-resistant PrP-A116V.	[79]
**Disease Type**	**PrPs**	**The Post-OR Sequence**	**Susceptibility to Prions**	**References**
Acquired prion disease	PrP∆32–80	Intact	• Fully susceptible to RML scrapie prions.	[80]
	PrP∆32–93	The post-OR residues 91–93 deleted	• Partially reduced to RML scrapie prions.	[81]
	PrP∆32–106	The post-OR residues 91–106 deleted	• Resistant to RML scrapie prions.	[82]

## Abbreviations

PrP	Prion protein
OR	Octapeptide repeat
WT	Wild-type
CJD	Creutzfeldt-Jakob disease
